# The invisible scars of emotional abuse: a common and highly harmful form of childhood maltreatment

**DOI:** 10.1186/s12888-021-03134-0

**Published:** 2021-03-17

**Authors:** Camila Monteiro Fabricio Gama, Liana Catarina Lima Portugal, Raquel Menezes Gonçalves, Sérgio de Souza Junior, Liliane Maria Pereira Vilete, Mauro Vitor Mendlowicz, Ivan Figueira, Eliane Volchan, Isabel Antunes David, Leticia de Oliveira, Mirtes Garcia Pereira

**Affiliations:** 1 Laboratório de Neurofisiologia do Comportamento (LABNEC), Departamento de Fisiologia e Farmacologia, Instituto Biomédico, Universidade Federal Fluminense, Niterói, Brazil; 2 Laboratório Integrado de Pesquisa em Estresse, Instituto de Psiquiatria, Universidade Federal do Rio de Janeiro, Av Venceslau Bras 71, Rio de Janeiro, 22290-140 Brazil; 3 Departamento de Psiquiatria e Saúde Mental, Universidade Federal Fluminense, Niterói, Brazil; 4 Laboratório de Neurobiologia, Instituto de Biofísica Carlos Chagas Filho, Universidade Federal do Rio de Janeiro, Av. Carlos Chagas Filho 373, Rio de Janeiro, 21941-902 Brazil

**Keywords:** Childhood maltreatment, Emotional abuse, Posttraumatic stress disorder, Revictimization

## Abstract

**Background:**

Childhood maltreatment (CM) is unfortunately widespread globally and has been linked with an increased risk of a variety of psychiatric disorders in adults, including posttraumatic stress disorder (PTSD). These associations are well established in the literature for some maltreatment forms, such as sexual and physical abuse. However, the effects of emotional maltreatment are much less explored, even though this type figures among the most common forms of childhood maltreatment. Thus, the present study aims to investigate the impact of each type of childhood maltreatment, both individually and conjointly, on revictimization and PTSD symptom severity using a nonclinical college student sample.

**Methods:**

Five hundred and two graduate and undergraduate students participated in the study by completing questionnaires assessing lifetime traumatic experiences in general, maltreatment during childhood and PTSD symptoms. Bivariate and multivariate negative binomial regressions were applied to examine the associations among childhood maltreatment, revictimization, and PTSD symptom severity.

**Results:**

Our results showed that using bivariate models, all types of CM were significantly associated with revictimization and PTSD symptom severity. Multivariate models showed that emotional abuse was the type of maltreatment associated with the highest incidence rates of revictimization and PTSD symptom severity.

**Conclusions:**

These data provide additional evidence of the harmful effects of childhood maltreatment and its long-term consequences for individuals’ mental health. Notably, the findings highlight the importance of studying the impacts of emotional abuse, which seems to be a highly prevalent, understudied, and chronic form of maltreatment that is as toxic as other maltreatment forms.

**Supplementary Information:**

The online version contains supplementary material available at 10.1186/s12888-021-03134-0.

## Background

Stressful experiences in childhood, especially those involving childhood maltreatment, began to be studied in the late 1970s and early 1980s [1]. Childhood maltreatment consists of abusive or neglectful acts perpetrated by parents or caregivers having the potential to “harm or threaten a child” [2]. Five subtypes of childhood maltreatment are commonly recognized: physical abuse, emotional abuse, sexual abuse, physical neglect and emotional neglect. In terms of prevalence, a worldwide meta-analysis estimated rates of 12.7% for sexual abuse, 16.3% for physical neglect, 18.4% for emotional neglect, 22.6% for physical abuse, and 36.3% for emotional abuse [3]. These data indicate that childhood maltreatment is globally widespread, affecting the lives of millions of children. Exposure to childhood maltreatment has been associated with a variety of psychiatric disorders in adults, such as depression and anxiety disorders [4], bipolar disorder [5, 6], eating disorders [7], personality disorders [8] and trauma-related disorders, such as posttraumatic stress disorder (PTSD) [9].

### Associations between childhood maltreatment and posttraumatic stress disorder

As a severe mental disorder that involves exposure to real or threatening death events, serious injury, or sexual violence, PTSD profoundly impairs cognitive and behavioural functioning. The main symptoms are reexperiencing, avoidance, negative mood and cognitions, and hyperarousal [10]. Trauma exposure is highly prevalent: epidemiological surveys suggest that approximately 70% of their samples reported lifetime exposure to at least one traumatic event [11, 12]. However, the prevalence of PTSD among the general population is less than 10% [13, 14]. Studies that explore the factors that might be related to an increased vulnerability to PTSD are crucial [15], and childhood maltreatment seems to be an important risk factor for PTSD development [16–21] and severity [9, 22–26].

The link between childhood physical and sexual abuse and PTSD is well established in the literature [24, 27–31], especially when investigated individually. However, studies exploring the impact of all childhood maltreatment types conjointly on PTSD symptomatology are sparse. Furthermore, childhood emotional maltreatment is much less explored as a potential vulnerability factor, not only to PTSD but also to psychiatric disorders in general (see [32] for a review). As mentioned before, it is important to highlight that emotional maltreatment not only figures among the most common forms of childhood maltreatment [3, 9, 23] but is also significantly associated with depressive symptoms [33–36], substance use disorders [37] and suicide risk [38, 39]. Nevertheless, emotional maltreatment rarely prompts specific actions for child protection. Thus, it is urgent to expand knowledge about the consequences of childhood emotional maltreatment, individually or conjointly with all other maltreatment types, on mental health. Particularly its role as a factor for PTSD vulnerability, considering the high prevalence of lifetime trauma exposure in the population [12] and the abundant evidence that other forms of maltreatment are a risk factor for this disorder.

### Associations between childhood maltreatment and Revictimization

Early caregiver-child relationships establish a critical foundation for lifelong learning and can have permanent sequelae. The lack of security in a maltreatment environment increases the risk for further trauma exposure [40, 41]. In fact, many studies have highlighted that childhood maltreatment is predictive of revictimization, which refers to the exposure of individuals who were victimized during childhood to subsequent traumatic events [42]. For instance, in a sample of substantiated childhood maltreatment victims, sexual and physical abuse experiences predicted revictimization [42, 43]. Similar results were found for male psychiatric inpatients [44] and in a community sample [45] for physical and sexual abuse. Emotional and sexual abuse during childhood predicted adult rape in college women [46]. Recently, a study suggested that all types of abuse and neglect, except for emotional neglect that was not investigated, were significantly associated with higher levels of revictimization in a sample of adolescent girls involved with the child welfare system [47]. Important differences in the characteristics of the samples used to probe the association between childhood maltreatment and revictimization, such as gender, age at investigation, and education level, make it more difficult to generalize the results to other populations. Dias et al. [23] was the only study that investigated the impact of all maltreatment types conjointly and found evidence that emotional abuse is significantly associated with revictimization and PTSD symptoms in a convenience sample from a European high-income country. Geographic and economic factors seem to play an important role in worldwide estimates of childhood maltreatment [48]. Thus, it is necessary to expand knowledge about how different forms of childhood maltreatment are related to revictimization and PTSD severity in other cultural contexts. Here, we explored the impact of all childhood maltreatment types on PTSD severity and revictimization in a nonclinical and relatively healthy sample from a South American middle-income country and exposed to a wide variety of forms of childhood maltreatment. According to Viola et al. [48], among all continents, South America has the highest rates of childhood maltreatment severity. Studies carried out with non-clinical samples present many advantages given that they reduce the biases of more severe cases, higher prevalence of psychiatric disorders, medication, and higher levels of functional impairment. For the purpose of this article, we consider a broader definition of revictimization, referring to individuals who were victimized during childhood and exposed to any subsequent type of traumatic event occurring during adolescence or adult life, not only a specific adverse experience.

In summary, the present study aims to investigate the impact of each type of childhood maltreatment, both individually and conjointly, on the severity of PTSD symptoms using a nonclinical Brazilian college student sample. Additionally, we explored the association of childhood maltreatment and revictimization. We hypothesize that those who experienced childhood maltreatment are more prone to experience other traumatic events and their harmful consequences and present higher levels of PTSD symptoms when facing another trauma later in life. We also hypothesize that all childhood maltreatment types, including emotional maltreatment, will impact mental health, predicting revictimization and PTSD symptoms for another trauma. Exploring the impact of emotional maltreatment is particularly relevant considering its high prevalence and the fact that it is the least visible form of maltreatment experienced by a child.

## Methods

### Participants

A sample of five hundred and two volunteers (mean age 21.2; SD = 4.01) participated in the survey. All participants were graduate or undergraduate students at Federal Fluminense University and at Federal University of Rio de Janeiro, Rio de Janeiro – Brazil. They were recruited through a brief announcement in their classrooms, and all interested students stayed in class and received numbered questionnaires. Then, they were instructed to read the consent terms, which guaranteed anonymity and freedom to end participation. After completing all the questionnaires, participants were instructed to put them into a box, with no individual identification.

The inclusion criterion was being 18 years old or older, and the only exclusion criterion was failing to fill out all the questionnaires. Fifty-nine participants who did not fully complete the questionnaire battery were excluded, leaving an “original sample” of 443 participants. The characteristics of this sample are described in Table 1. This was the sample used for revictimization analysis.

**Table 1:** Characteristics of the Original Sample

Sample characteristics	n	Mean (SE)/%
**Age – years (18–52 years)**	443	21.2 (0.2)
**Sex**		
Female	350	79
Male	93	21
**Childhood Maltreatment (CM)**		
Without CM	115	26
At least one CM	328	74
Physical Abuse	132	29.8
Sexual Abuse	94	21.2
Emotional Abuse	260	58.7
Physical Neglect	92	20.8
Emotional Neglect	187	42.2
Two or more CM	223	50.3
**Mean quantity of types of traumatic events after 12 years (SE)**	443	5.6 (0.1)

This study was approved by the Ethics Review Board of the Federal University of Rio de Janeiro, process number CAAE 56431116.5.0000.5263, and all methods were carried out in accordance with relevant guidelines and national regulations. Each participant gave written informed consent prior to participation.

### Measures

#### Trauma History Questionnaire (THQ)

Translated and adapted to Portuguese [49] from the original [50], the Trauma History Questionnaire (THQ) is a self-report questionnaire that examines exposure to different types of traumatic events, from urban violence crimes to sexual assault and natural disasters. The scale is composed of 23 items divided into three clusters (crime-related events, trauma and disaster in general, and sexual and physical experiences) that investigate potentially traumatic events through yes/no questions and further investigate frequency and approximate age at the time of exposure. The questionnaire also contains an open-ended question that allows participants to specify other extraordinarily stressful situations or events that they have experienced.

In this study, one subitem was added to all the questionnaire items to determine the intensity of the worst event (0 = not stressful at all; 5 = extremely stressful). For all the analyses involving the THQ, we included only the traumatic events that occurred after 12 years of age (i.e., after childhood according to local laws and the NIH definitions cited above) and with an intensity score ≥ 3 (mild to extremely stressful). The test-retest reliability results in a psychometric evaluation study of trauma and PTSD indicated moderate to high coefficients [51].

#### Posttraumatic stress disorder checklist for DSM-5 (PCL-5)

Posttraumatic stress symptoms were assessed using the PCL-5, which was developed by the National Center for PTSD in accordance with the DSM-5 [10, 52]. Translated and adapted to Portuguese [53], the PCL-5 is a 20-item self-report questionnaire that measures the four cluster symptoms of PTSD: intrusion, avoidance, negative alterations in cognition and mood, and alterations in arousal and reactivity. Each item in the PCL-5 questionnaire is rated on a 5-point Likert scale (from 0= “Not at all” to 4 = “Extremely”). Symptom severity can be calculated by summing the items in each of the four clusters or summing all 20 items. In this case, the severity score ranges from zero to 80 points. For our study, we opted to consider the total score to analyse symptom severity [52]. Participants were instructed to consider one worst event previously reported in the THQ, as they indicated how each item of the PCL-5 bothered them in the last month.

The psychometric properties of the PCL-5 have been assessed in different cultural contexts and samples, presenting satisfactory to high internal consistency, very good to high test-retest reliability and strong convergent and discriminant validity [54–57].

#### Childhood Trauma Questionnaire - Short Form (CTQ-SF)

Childhood maltreatment (CM) was quantitatively assessed with the 28-item Childhood Trauma Questionnaire (CTQ) [58, 59] that was translated and adapted to Portuguese [60]. It measures childhood exposure to physical, emotional and sexual abuse and physical and emotional neglect. The instrument has five items exploring each of these five subtypes of CM, yielding 25 items for analysis and three more items to investigate minimization and denial. Participants respond to each item on a scale from 1 (“Never”) to 5 (“Always”), which indicates the frequency with which they had these experiences. Responses are converted into a maltreatment severity subtype: “None to Minimal”, “Low to Moderate”, “Moderate to Severe” or “Severe to Extreme” [58].

We used the Bernstein and Fink [58] cut-off points for “Low to Moderate” severity to classify the presence of CM (physical abuse≥8; sexual abuse ≥6; emotional abuse ≥9; physical neglect ≥8; emotional neglect ≥10) [58]. Thus, the presence of maltreatment was considered if a participant had a CTQ score equal to or higher than the low to moderate cut-off point for each maltreatment type.

#### Revictimization

We considered revictimization as any subsequent type of traumatic event occurring during adolescence or adult life in victims of childhood maltreatment. Revictimization was measured by summing the quantity of types of traumatic events reported in the THQ with an intensity greater than or equal to 3 that occurred after the age of 12 years. Thus, events that met these criteria were summed and provided a final revictimization score for each volunteer.

**Fig. 1:**
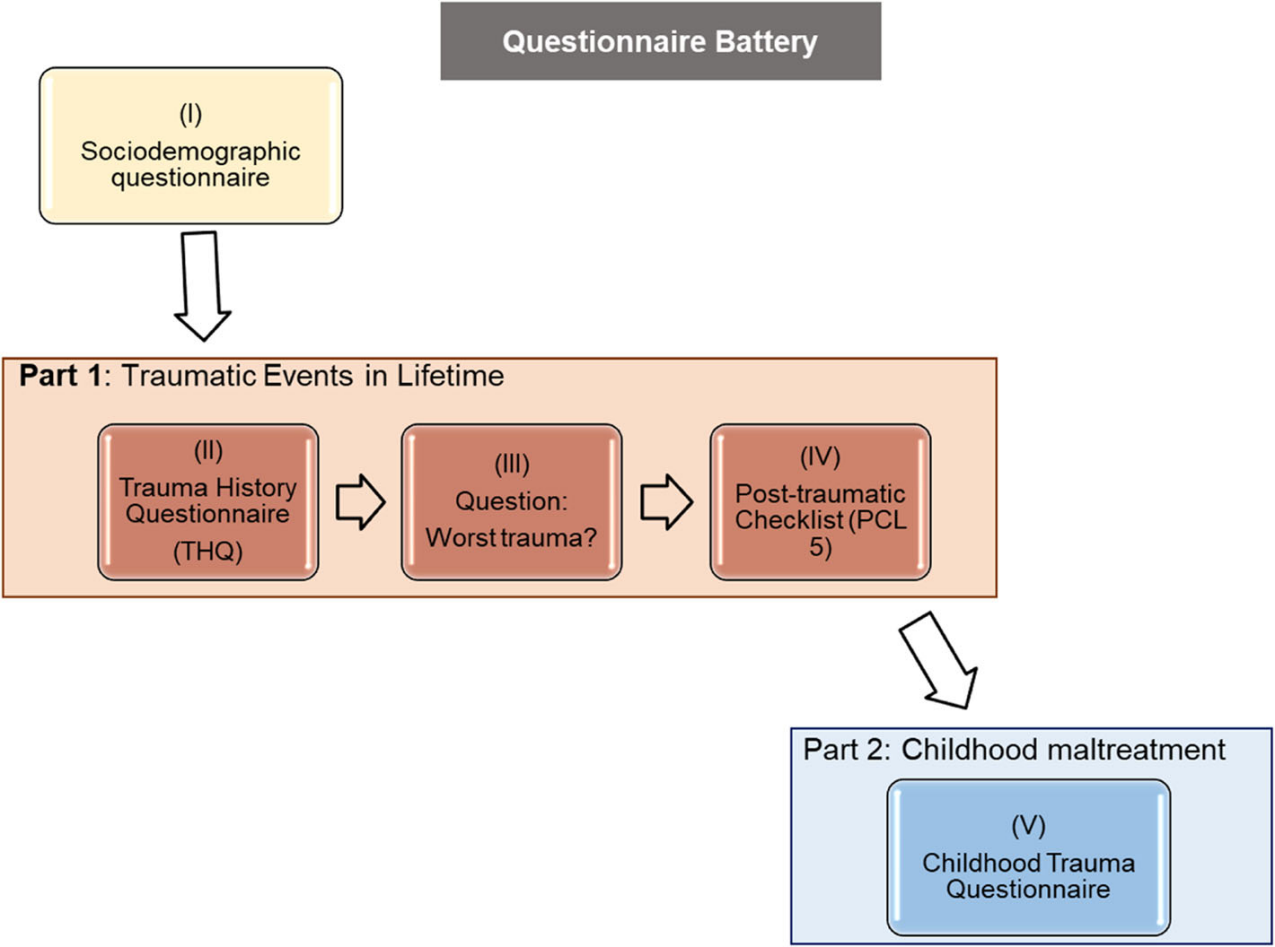
Diagram describing the specific order of the scales in the questionnaire battery. The basic sociodemographic questionnaire was the first presented, succeeded by two parts. Part one was composed of the Trauma History Questionnaire (THQ) [49, 50] and Posttraumatic Stress Disorder Checklist 5 (PCL-5) [52, 53], and part two contained the Childhood Trauma Questionnaire (CTQ) [58–60]. At the end of the THQ, participants had to indicate the event that they considered the most traumatic in their life and answer the PCL-5 based on this event

It is important to mention that we assessed childhood maltreatment using the CTQ scale and that participants were instructed to answer the questionnaire based only on their childhood experiences (not including adolescence or adult life). Childhood is locally defined as the period before the age of 12 years (Brazil, Law 8069 - Child and Adolescence Statute) [61, 62]. Additionally, questionnaires investigating childhood versus later periods of life were presented in different parts in the questionnaire booklet (see Fig. 1 and procedures subsection). The instruction to consider only events that occurred during childhood was reinforced in the beginning of the second part (Childhood maltreatment part). When measuring revictimization, we considered only traumas occurring above the age of 12 years in the THQ. This procedure was important to avoid an overlap between events considered childhood maltreatment and those included in revictimization scores.

To investigate the association between childhood maltreatment and revictimization, we used the original sample of 443 participants.

#### Childhood maltreatment and PTSD severity

Additional exclusion criteria were applied to investigate the effects of different forms of childhood maltreatment on the prediction of PTSD severity for a subsequent trauma (that occurred during adolescence or adulthood). Participants were excluded if they did not report an index traumatic event in one of the Trauma History Questionnaire clusters (see the Measures section below) or if the index traumatic event reported occurred before 12 years of age (*n* = 181). The rationale was to include only participants with an index trauma that met PTSD criterion A and that occurred after 12 years of age. This age cut-off was set to guarantee that the PTSD symptoms were related to the index for trauma that occurred after childhood, following local definitions of the age range for childhood [61, 62].

Thus, the final sample for the analysis that examined the influence of childhood maltreatment on PTSD severity (“PTSD symptoms sample”) to a subsequent trauma comprised 262 volunteers. Note that this additional exclusion was applied exclusively to the PTSD symptom severity analysis.

### Procedure

The questionnaires were distributed in classrooms, and volunteers took approximately 1 h and 20 min to complete them. Each questionnaire was composed of a self-report basic sociodemographic survey collecting data on sex, age, religion, educational level, family income and previous and current diagnosed disorders, followed by three self-report scales. As shown in Fig. 1, the scales were grouped into two parts. In the first part, participants were instructed to complete the questionnaires in accordance with their lifetime experiences. Volunteers completed the (I) THQ and (II) PCL-5. At the end of the THQ, participants were asked to indicate the event that they considered the most traumatic in their life. Participants answered the PCL-5 based on the traumatic event identified as the worst in the THQ. In the second part, participants were instructed to fill out the questionnaire according to their childhood experiences and completed the (III) Childhood Trauma Questionnaire - Short Form (CTQ-SF). They were asked to report responses on as many experiences as they could remember.

### Statistical analysis

First, we calculated the average age of the participants and the proportions for sex, absence of any type of childhood maltreatment, presence of at least one type of childhood maltreatment and presence of each type of childhood maltreatment. We considered maltreatment as present if the participant had a CTQ score equal to or higher than the “low to moderate” cut-off point, according to Bernstein and Fink’s [58] cut-off points, for each maltreatment type. The average quantity of traumatic events according to the THQ self-reports was also computed.

Normality tests were carried out to investigate the distribution profile of the dependent variables. The Shapiro–Wilk test indicated that the number of types of traumatic events and the PCL-5 scores did not follow a normal distribution (quantity of traumatic events: W = 0.97; *p* < 0.000; PCL-5: W = 0.92; *p* < 0.000).

Negative binomial models were used to address the problem of overdispersed count data. The exponentiated regression coefficients provide the incidence ratio, which is interpreted as an increase or decrease in the dependent variable in terms of percentage for each unit change of the independent variable. We performed bivariate and multivariate negative binomial regressions to examine the influence of childhood maltreatment with respect to two outcomes: revictimization (measured as the quantity of types of traumatic events after childhood, i.e., 12 years old, with an intensity score ≥ 3 reported in the THQ) and PTSD severity for a subsequent trauma (PCL-5 score based on the worst traumatic event reported in one of the THQ clusters and that occurred after childhood, i.e., 12 years old). Age, gender and socioeconomic status were included as potential confounders in the multivariate models.

The independent variable of interest was the presence of childhood maltreatment reported in the CTQ, and the dependent variables were the number of types of traumatic events after 12 years old reported in the THQ and the PCL-5 score for the worst traumatic event. In the modelling processes for the two outcomes, we followed the same strategy. First, we performed bivariate analysis to examine the influence of each form of childhood maltreatment on the prediction of revictimization or PTSD severity later in life. The forms with *p*-values less than 0.20 and with confidence intervals that did not present a null value (i.e., CI did not include 1.0) were selected for inclusion in the multivariate model. Those with p-values less than 0.10 and with confidence intervals that did not present a null value were retained in the model.

All statistical analyses were performed using the Stata 12.0 package, and statistical significance was established at *p* < 0.05.

## Results

### Childhood maltreatment and revictimization

#### Original sample characteristics

**Fig. 2:**
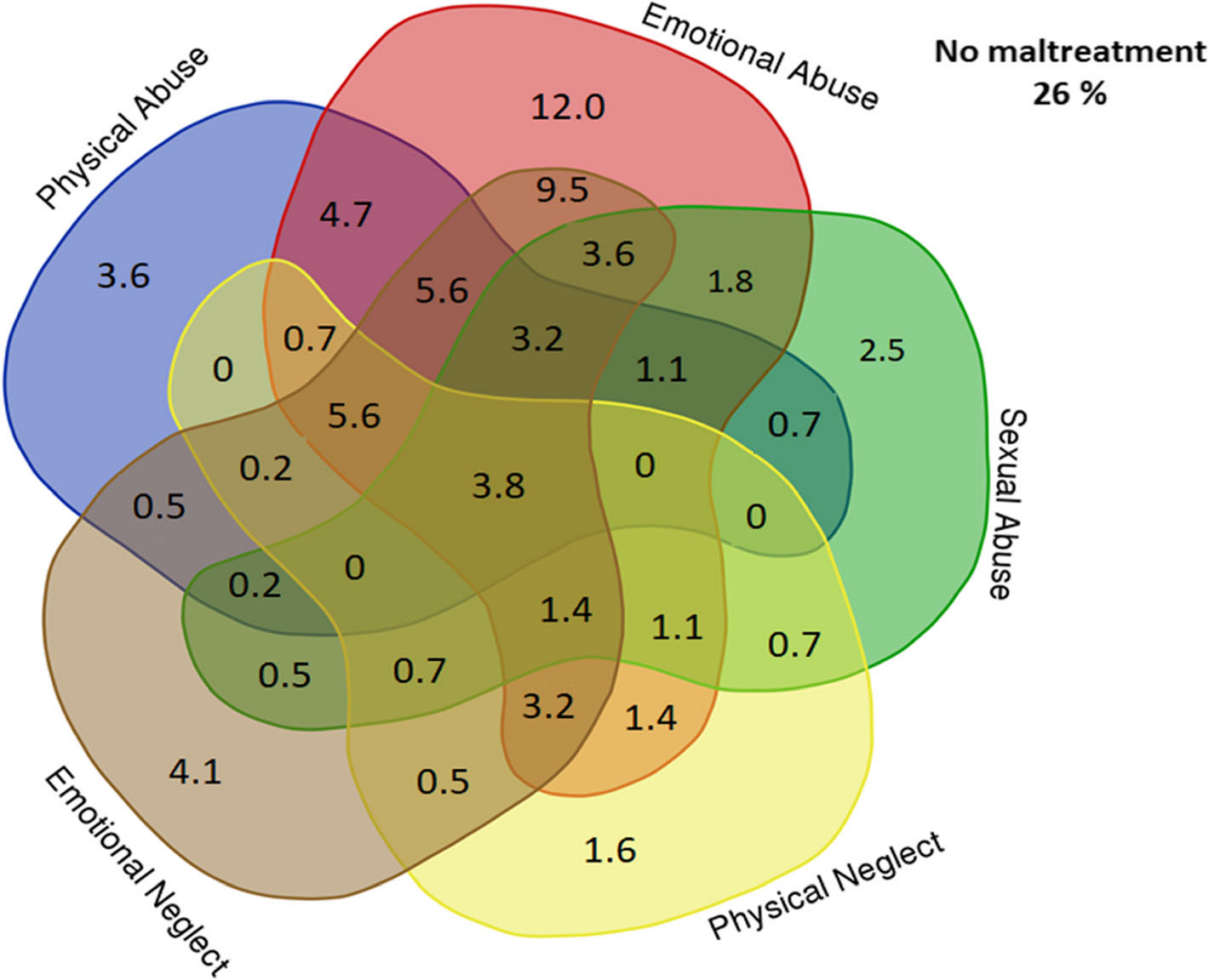
Venn diagram illustrating the percentage of single types and the overlap of types of childhood maltreatment in the original sample (*n* = 443). This diagram was partially constructed using an online tool available at (http://bioinformatics.psb.ugent.be/cgi-bin/liste/Venn/calculate_venn.htpl)

Information on participants’ age, sex, childhood maltreatment exposure, and quantity of types of traumatic events is provided in Table 1. As shown, the original sample (*N* = 443) was mainly female (79%), and 74% reported the presence of at least one type of childhood maltreatment. Emotional abuse and emotional neglect presented the highest frequencies of exposure. The mean number of types of traumatic events that occurred after 12 years of age was 5.6 (SE = 0.1). Furthermore, Fig. 2 and Table 2 depicts the percentage of volunteers from the original sample who reported a single type of childhood maltreatment as well as the overlap between the maltreatment types. Overall, the co-occurrence of different types of maltreatment was common. For emotional abuse, we observed a slightly higher percentage of participants who reported a single type of maltreatment.

**Table 2:** Percentage of single types and the overlap of types of childhood maltreatment in the original sample (*n* = 443)

	Single Maltreatment (%)	Two Maltreatments	%	Three Maltreatments	%	Four Maltreatments	%
**Emotional Abuse**	12	+ Sexual Abuse	1.8	+ Sexual Abuse + Physical Abuse	1.1	+Sexual Abuse + Physical Abuse + Emotional Neglect	3.2
		+ Physical Abuse	4.7	+ Sexual Abuse + Emotional Neglect	3.6	+Sexual Abuse + Physical Abuse + Physical Neglect	0
		+ Emotional Neglect	9.5	+ Sexual Abuse + Physical Neglect	1.1	+Sexual Abuse + Emotional Neglect + Physical Neglect	1.4
		+ Physical Neglect	1.4	+ Physical Abuse + Emotional Neglect	5.6	+Physical Abuse + Emotional Neglect + Physical Neglect	5.6
				+ Physical Abuse + Physical Neglect	0.7		
				+ Emotional Neglect + Physical Neglect	3.2		
**Sexual Abuse**	2.5	+ Emotional Abuse	1.8	+ Emotional Abuse + Physical Abuse	1.1	+ Emotional Abuse + Physical Abuse + Emotional Neglect	3.2
		+ Physical Abuse	0.7	+ Emotional Abuse + Emotional Neglect	3.6	+ Emotional Abuse + Physical Abuse + Physical Neglect	0
		+ Emotional Neglect	0.5	+ Emotional Abuse + Physical Neglect	1.1	+ Emotional Abuse + Emotional Neglect + Physical Neglect	1.4
		+ Physical Neglect	0.7	+ Physical Abuse + Emotional Neglect	0.2	+ Physical Abuse + Emotional Neglect + Physical Neglect	0
				+ Physical Abuse + Physical Neglect	0		
				+ Emotional Neglect + Physical Neglect	0.7		
**Physical Abuse**	3.6	+ Emotional Abuse	4.7	+Emotional Abuse + Sexual Abuse	1.1	+ Emotional Abuse + Sexual Abuse + Emotional Neglect	3.2
		+ Sexual Abuse	0.7	+ Emotional Abuse + Emotional Neglect	5.6	+ Emotional Abuse + Sexual Abuse + Physical Neglect	0
		+ Emotional Neglect	0.2	+ Emotional Abuse + Physical Neglect	0.7	+ Emotional Abuse + Emotional Neglect + Physical Neglect	5.6
		+ Physical Neglect	0	+ Sexual Abuse + Emotional Neglect	0.2	+ Sexual Abuse + Emotional Neglect + Physical Neglect	0
				+ Sexual Abuse + Physical Neglect	0		
				+ Emotional Neglect + Physical Neglect	0.2		
**Emotional Neglect**	4.1	+ Emotional Abuse	9.5	+ Emotional Abuse + Sexual Abuse	3.6	+ Emotional Abuse + Sexual Abuse + Physical Abuse	0
		+ Sexual Abuse	0.5	+ Emotional Abuse + Physical Abuse	5.6	+ Emotional Abuse + Sexual Abuse + Emotional Neglect	1.4
		+ Physical Abuse	0.2	+ Emotional Abuse + Physical Neglect	3.2	+ Emotional Abuse + Physical Abuse + Emotional Neglect	5.6
		+ Physical Neglect	0.5	+ Sexual Abuse + Physical Abuse	0.2	+ Sexual Abuse + Physical Abuse + Emotional Neglect	0
				+ Sexual Abuse + Physical Neglect	0.7		
				+ Physical Abuse + Physical Neglect	0.2		
**Physical Neglect**	1.6	+ Emotional Abuse	1.4	+ Emotional Abuse + Sexual Abuse	1.1	+ Emotional Abuse + Sexual Abuse + Physical Abuse	0
		+ Sexual Abuse	0.7	+ Emotional Abuse + Physical Abuse	0.7	+ Emotional Abuse + Sexual Abuse + Emotional Neglect	1.4
		+ Physical Abuse	0	+ Emotional Abuse + Emotional Neglect	3.2	+ Emotional Abuse + Physical Abuse + Emotional Neglect	5.6
		+ Emotional Neglect	0.5	+ Sexual Abuse + Physical Abuse	0	+ Sexual Abuse + Physical Abuse + Emotional Neglect	0
				+ Sexual Abuse + Emotional Neglect	0.7		
				+ Physical Abuse + Emotional Neglect	0.2		
**Five maltreatments**	3.8						
**No maltreatment**	26						

For mean CTQ total scores and subscales scores see supplemental material Table S1.

### Frequency of traumatic events occurring during adolescence/adulthood in the original sample

The percentage of volunteers who reported an intensity of three or higher for at least one question in each of the THQ clusters is presented in Table 3. Note that the same volunteer can report an intensity of three or higher for questions in more than one cluster.

**Table 3:** Frequency of traumatic events for each THQ cluster

THQ cluster	N	%
**Crime-related events** (e.g., something being taken directly from you by force or someone attempting to rob you)	289	65.2
**Trauma and disaster in general** (e.g., a serious accident at work, a natural disaster or the death of someone close to you)	398	89.8
**Sexual and physical experiences** (e.g., having intercourse or oral or anal sex against your will or witnessing it happen to family members or riends, being attacked with a gun or knife)	143	32.3

The highest rate of exposure was found for the “Trauma and disaster in general” cluster, with almost 90% of the sample reporting at least one event in this cluster, followed by 65.2% of the sample reporting at least one event in the “Crime-related events” cluster and 32.3% in the “Sexual and physical experiences” cluster.

Only traumas reported as occurring at ≥12 years old were included. The percentage was calculated by dividing the number of volunteers who reported an intensity of 3 or higher for at least one item in each cluster by the number of volunteers in the original sample.

### Predicting revictimization

We used the original sample to investigate the influence of different forms of childhood maltreatment on the prediction of revictimization (number of types of traumatic events after childhood reported in the THQ). We performed five bivariate negative binomial regressions to investigate whether each type of childhood maltreatment was associated with revictimization (number of types of traumatic events occurring after childhood). As shown in Table 4 (bivariate model), all types of childhood maltreatment caused an increase in the incidence of revictimization. Note that emotional abuse had the highest impact in this sample, causing a 52% increase in the incidence rate of revictimization for participants who were exposed to this maltreatment compared to those who were not exposed. In other words, participants exposed to emotional abuse during childhood had on average 52% more types of traumatic events (with an intensity rating of 3 or higher) that occurred after 12 years of age.

**Table 4:** The Impact of Childhood Maltreatment on Revictimization: Bivariate and Multivariate Negative Binomial Regression Model

Revictimization	Bivariate Model			Multivariate Model (raw)			Multivariate Model (adjusted)		
	*IRR*	95% CI	*p-value*	*IRR*	95% CI	*p-value*	*IRR*	95% CI	*p-value*
Physical Abuse	1.25	[1.10–1.41]	0.000	1.05	[0.93–1.19]	0.408	1.04	[0.92–1.17]	0.536
Sexual Abuse	1.33	[1.17–1.52]	0.000	1.20	[1.05–1.36]	0.006	1.14	[1.01–1.30]	0.041
Emotional Abuse	1.52	[1.35–1.70]	0.000	1.40	[1.23–1.59]	0.000	1.40	[1.23–1.59]	0.000
Physical Neglect	1.30	[1.14–1.49]	0.000	1.12	[0.97–1.28]	0.115	1.09	[0.95–1.25]	0.200
Emotional Neglect	1.29	[1.15–1.44]	0.000	1.03	[0.91–1.17]	0.648	1.03	[0.91–1.17]	0.620

When all the maltreatment forms were included in the same model but without controlling for confounders, only sexual and emotional abuse significantly predicted the risk for revictimization (Table 4 – raw multivariate model). When gender, age and socioeconomic status were included as potential confounders, sexual (*p* < 0.041, 95% CI [1.01–1.30]) and emotional abuse (*p* < 0.000, 95% CI [1.23–1.59]) remained statistically significant (Table 4 - adjusted multivariate model). Emotional abuse showed the highest impact in this sample, causing a 40% increase in the average number of types of subsequent (after 12 years old) traumatic events.

### Childhood maltreatment and PTSD symptom severity

#### Characteristics of the PTSD symptom sample

The association between the occurrence of childhood maltreatment and PTSD symptom severity for a subsequent trauma was investigated in a subsample of participants, the PTSD symptom sample. For this analysis, only the participants who answered the PCL-5 based on a traumatic event reported in one of the THQ clusters that occurred after childhood (after 12 years old) were included (262 volunteers). Table 5 shows the characteristics of this sample in terms of age, sex, childhood maltreatment exposure, and mean PTSD score. Note that for this subsample, emotional abuse and emotional neglect also presented the highest frequencies of occurrence among the maltreatment types. The frequency of lifetime traumatic events considered the most traumatic and used to answer the PCL-5 is shown in Table 6.

**Table 5:** Characteristics of the PTSD Symptom Sample

Sample characteristics	n	Mean (SE)/%
**Age – years (18 - 52 years)**	262	21.3 (0.3)
**Sex**		
Female	215	82.1
Male	47	17.9
**Childhood Maltreatment (CM)**		
Without CM	79	30.2
At least one CM	183	69.8
Physical Abuse	69	26.3
Sexual Abuse	41	15.6
Emotional Abuse	140	53.4
Physical Neglect	49	18.7
Emotional Neglect	98	37.4
Two or more CM	115	43.9
**Posttraumatic Stress Symptom Severity**	262	19.1 (1.0)

**Table 6:** Frequency of the worst traumatic events by THQ cluster

THQ cluster	Worst trauma	
	N	%
**Crime-related events** (e.g., something being taken directly from you by force or someone attempting to rob you)	47	17.9
**Trauma and disaster in general** (e.g., a serious accident at work, a natural disaster or the death of someone close to you)	169	64.5
**Sexual and physical experiences** (e.g., having intercourse or oral or anal sex against your will or witnessing it happen to family members or friends, being attacked with a gun or knife)	46	17.6

#### Predicting PTSD symptom severity

To investigate the association between each type of childhood maltreatment and PTSD symptom severity for a subsequent trauma, we ran five bivariate negative binomial regressions. As shown in Table 7 (bivariate model), all types of childhood maltreatment significantly predicted PTSD severity for a subsequent trauma. These results show that all forms of childhood maltreatment caused an increase in the average PCL-5 scores for another trauma occurring in adolescence/adulthood. Note that emotional abuse had the highest impact, being associated with an increase of 94% in the incidence rate of PTSD symptoms.

**Table 7:** Predicting PTSD Symptoms: Bivariate and Multivariate Negative Binomial Regression for the Prediction of PTSD Severity

PTSD	Bivariate Model			Multivariate Model (raw)			Multivariate Model (adjusted)		
	*IRR*	95% CI	*p-value*	*IRR*	95% CI	*p-value*	*IRR*	95% CI	*p-value*
Physical Abuse	1.33	[1.03–1.72]	0.026	1.01	[0.79–1.30]	0.911	1.00	[0.78–1.28]	0.981
Sexual Abuse	1.80	[1.33–2.42]	0.000	1.56	[1.16–2.09]	0.003	1.48	[1.10–1.99]	0.010
Emotional Abuse	1.94	[1.56–2.40]	0.000	1.76	[1.39–2.22]	0.000	1.77	[1.40–2.23]	0.000
Physical Neglect	1.72	[1.30–2.28]	0.000	1.34	[1.00–1.79]	0.052	1.29	[0.96–1.73]	0.087
Emotional Neglect	1.48	[1.18–1.86]	0.001	0.96	[0.74–1.23]	0.729	0.96	[0.75–1.23]	0.756

When all the childhood maltreatment forms were entered in the same model, emotional and sexual abuse remained significant predictors of PTSD severity for a subsequent trauma (that occurred after childhood), causing increments of 76 and 56%, respectively, in the incidence rate of PTSD symptoms (Table 7 – raw multivariate model). Physical neglect was associated with a more moderate effect. When control variables (age, gender and socioeconomic status) were included in the model, emotional abuse and sexual abuse remained significant, and emotional abuse still showed the highest impact in this sample, causing a 77% increase in the average posttraumatic stress symptoms (Table 7 – adjusted multivariate model).

For completeness, the results of the bivariate and multivariate models for revictimization and PTSD symptom prediction, but considering CTQ scores as continuous variables, are presented in the supplemental material (Tables S2 and S3 respectively).

## Discussion

This study aimed to investigate whether the presence of childhood maltreatment, especially emotional abuse maltreatment, could predict revictimization and PTSD severity symptoms for a subsequent traumatic event in adolescence and young adulthood. Our main results demonstrate that each maltreatment subtype, when individually analysed in bivariate regressions, was significantly associated with revictimization and with PTSD symptom severity. Moreover, when all the forms of maltreatment were investigated together in a multivariate regression model, emotional and sexual abuse remained significant predictors of revictimization and PTSD severity symptoms. Importantly, emotional abuse was associated with the largest increases in the number of types of subsequent traumatic events and the highest incident rates of PTSD symptoms, highlighting the long-term consequences of emotional maltreatment in a nonclinical sample of Brazilian college students.

In addition, 74% of our sample was exposed to at least one form of childhood maltreatment, and 50.3% of students reported being exposed to two or more types of childhood maltreatment. In fact, among all continents, South America, and specifically Brazil, has been reported to have the highest rates of estimated childhood maltreatment [48]. Additionally, in our sample, emotional maltreatment was the most common form of maltreatment, with prevalence rates of 59 and 42% for emotional abuse and emotional neglect, respectively. These results are in line with a meta-analysis of worldwide prevalence that showed that emotional abuse is a universal problem [3] and with a previous study by Grassi-Oliveira and Stein [9] that also showed that emotional abuse was the most prevalent childhood maltreatment type in a low-income Brazilian sample.

In line with the literature, our data revealed that childhood maltreatment is associated with revictimization. Individuals who were victimized during their childhood reported a higher number of types of traumatic events that occurred later during adolescence/adulthood. The association of childhood maltreatment and revictimization was present for each subtype of maltreatment when analysed individually, but only emotional and sexual abuse remained significant predictors for revictimization when all subtypes were included in the same regression model. Consistent with our findings, other studies also reported that particular types of childhood maltreatment are associated with subsequent revictimization [42–47]. However, the majority of the studies focused primarily on childhood physical and sexual abuse, including substantiated cases, and/or did not investigate all five types of childhood maltreatment reported here. One exception is the study by Dias et al. [23], which also explored all forms of maltreatment and found that individuals who experienced emotional or physical abuse had higher risks for revictimization than those who did not.

Remarkably, in our sample, emotional abuse was the maltreatment subtype that showed the highest impact, causing an increase of 40% in the average number of types of subsequent traumatic events reported by individuals. In fact, if CTQ scores for each maltreatment are considered a continuous independent variable in the multivariate model for revictimization prediction, instead of a categorical presence/absence variable, emotional abuse is the only significant predictor of revictimization after controlling for potential confounders (see supplemental material). One possible explanation of why adverse situations related to childhood maltreatment lead to revictimization is that experiencing these events impairs the cognitive processing of emotional situations and compromises the acquisition of emotional-regulation capacities and interpersonal skills [63]. In fact, it was demonstrated that trauma exposure during childhood impairs neural processing of salient emotional stimuli and is associated with a failure to differentiate between nonthreat and threat-related stimuli [64]. Interestingly, Burns and colleagues [65] showed that emotional abuse was strongly related to emotional regulation difficulties, suggesting that emotion regulation skills might be more likely to be negatively impacted by emotional abuse than by other forms of maltreatment due to the former’s more chronic nature.

In addition to diminished risk detection skills, childhood maltreatment may lead to long-term dysregulation of the functioning of biological stress responses and hamper the implementation of typical defensive responses at imminent risk of victimization [66]. Arguments of dysfunction in the brain’s normal fear/defence circuit and impaired defensive engagement due to cumulative traumatization were also proposed by Lang and McTeague [67]. In their study, PTSD patients who had experienced recurrent traumatic exposure were among the least reactive to emotional stimuli and often reported a history of repeated childhood maltreatment exposure. It is important to mention that revictimization involves many other aspects in addition to the individual difficulties mentioned above. Interpersonal and sociocultural factors certainly contribute to an increased risk of experiencing other traumatic events. For example, cultural patterns and belief systems (ex. rigid gender roles) tend to create an environment that puts the victim in an unprotected situation, which in turn facilitates revictimization. In addition, family of origin functioning, characteristics of the initial maltreatment (ex. frequency, age of onset), community (ex. lack of family support), lack of resources, lack of security in a maltreatment environment and practices that normalize victim blaming were also identified as risk factors for further trauma exposure [40, 41, 68, 69].

Additionally, experiencing adverse situations during childhood has been consistently identified as a potential risk factor for mental health problems, including PTSD. Indeed, our results showed that all maltreatment subtypes, when individually analysed in bivariate regression models, were significantly associated with an increase in PTSD symptoms. This finding is consistent with several previous studies reporting that childhood maltreatment is associated with increased PTSD symptoms [9, 22–26]. Moreover, we showed that when all the forms of maltreatment were investigated together in a multivariate regression model, emotional and sexual abuse remained significant predictors of PTSD severity symptoms. There is abundant evidence confirming the negative consequences of sexual abuse during childhood [28, 32, 70, 71], but much less attention is given to emotional abuse. Remarkably, emotional abuse was the form of maltreatment that caused the highest increase in the incidence rate of PTSD symptoms in our sample. In the same vein, it was also reported that emotional abuse had the largest effect on the prediction of PTSD severity [23] and psychological symptoms [72] in a Portuguese community sample.

One of the most important symptoms of PTSD is the re-experiencing of the traumatic event, which has been linked to an inability to downregulate negative emotions [73], an overreaction to and a failure to recover from unpleasant events [74–77]. Accordingly, increased brain reactivity to negative stimuli [78] and difficulty with emotion regulation [79] were related to posttraumatic stress symptom severity in trauma-exposed undergraduate students. One of the pathways by which childhood maltreatment might lead to increased risk for PTSD is that childhood maltreatment could cause impairments in the ability to understand and regulate emotions, and emotional abuse in particular emerged as the strongest predictor of emotion dysregulation [65]. As emphasized by this study, emotional abuse usually occurs more frequently than other forms of maltreatment, and this might overwhelm an individual’s capacity to effectively regulate emotions, as he or she is chronically exposed to situations involving negative affect [65]. Taken together, these findings support the urgent need to identify and treat individuals who suffer emotional maltreatment due to its high probability of being associated with poor mental health in adulthood.

### Limitations

This study presents some limitations. As a cross-sectional study, the retrospective design may have led to recall bias. Individuals were asked to report PTSD symptoms based on their worst trauma, and only individuals who reported an index trauma that occurred after childhood were included in the analysis. However, considering the youthfulness of the participants, it is conceivable that the posttraumatic symptomatology reported is based on both childhood and adult traumas and not solely on adult trauma. It is also important to keep in mind that the co-occurrence of different types of childhood maltreatment might have influenced our results. Furthermore, the sample was predominantly female, which might have inflated our results, as recent studies have shown that the prevalence of emotional abuse is higher for women than for men [34, 80, 81]. All sources of data were obtained using the same method, self-report questionnaires, which could lead to common method variance. Strategies such as creating a psychological separation among measurements, protecting the anonymity of the respondents, and minimizing evaluation apprehension were carefully implemented in our procedures to minimize this problem [82]. The external validity of the findings is limited due to the homogeneity of the present sample, and the results might not be generalizable to clinical populations. Nevertheless, this study can provide important insights into how harmful untimely experiences can be in a traumatized young student sample. In addition, the homogeneity of this sample may suggest that similar results could be found for samples with similar characteristics.

## Conclusions

In sum, this study provides additional knowledge on the harmful effects of childhood maltreatment and its long-term consequences for individuals’ mental health. Particularly, it highlights the importance of studying the consequences of emotional abuse, which seems to be a universal and chronic form of maltreatment that has a strong impact across the lifespan and that may be more harmful than other types of maltreatment. Emotional abuse needs to be studied further, and research on it has lagged behind that on other forms of childhood maltreatment. One key aspect of emotional abuse research is its lack of consideration in the diagnosis of PTSD. Considering that the concept of trauma encompasses different traumatic experiences not previously considered traumas but that are also harmful, future reformulations of the definition of traumatic events could contemplate emotional abuse.

In addition, the focus on emotional abuse might encourage the development of prevention and treatment strategies. By understanding how implicit memories of emotional abuse episodes impact future emotional regulation capacity, we might prevent the harmful effects of this type of abuse on intergenerational attachment styles, which can lead to societal problems such as parental violence, marital violence, and mental health disorders. Accordingly, the improvement of intervention strategies for memory reconsolidation and reprocessing of those events could have an immense impact on society.

## Supplementary Information

**Additional file 1: Table S1.** Mean CTQ total scores and subscales scores (original sample). **Table S2.** The Impact of Childhood Maltreatment on Revictimization: Bivariate and Multivariate Negative Binomial Regression Model (using CTQ scores) . **Table S3.** Predicting PTSD Symptoms: Bivariate and Multivariate Negative Binomial Regression for the Prediction of PTSD Severity (using CTQ scores).

## Data Availability

The datasets generated and/or analysed during the current study are not publicly available due to local ethics committee restrictions but are available from the corresponding author on reasonable request.

## Abbreviations

CI: Confidence Interval
CM: Childhood maltreatment
CTQ-SF: Childhood Trauma Questionnaire - Short Form
IRR: Incidence Rate Ratio
N: Sample number
PCL-5: Posttraumatic Stress Disorder Checklist for DSM-5
PTSD: Posttraumatic Stress Disorder
SE: Standard error
THQ: Trauma History Questionnaire
W: Shapiro-Wilk Test
