# Supplementary material for: The invisible scars of emotional abuse: a common and highly harmful form of childhood maltreatment
Source: BMC Psychiatry. 2021 Mar 17;21:156. doi: 10.1186/s12888-021-03134-0 (PMC7968325; doi:10.1186/s12888-021-03134-0)
Supplement: Supplementary file 1 — Additional file 1: Table S1. Mean CTQ total scores and subscales scores (original sample). Table S2. The Impact of Childhood Maltreatment on Revictimization: Bivariate and Multivariate Negative Binomial Regression Model (using CTQ scores) . Table S3. Predicting PTSD Symptoms: Bivariate and Multivariate Negative Binomial Regression for the Prediction of PTSD Severity (using CTQ scores). [file 12888_2021_3134_MOESM1_ESM.docx]

**Title page**

**The invisible scars of emotional abuse: a common and highly harmful form of childhood maltreatment**

Camila Monteiro Fabricio Gamaᵃ, Liana Catarina Lima Portugalᵃ, Raquel Menezes Gonçalvesᵃ, Sérgio de Souza Juniorᵃ, Liliane Maria Pereira Vileteᵇ, Mauro Vitor Mendlowiczᵃᵇ, Ivan Luiz de Vasconcellos Figueiraᵇ, Eliane Volchanᵇ, Isabel de Paula Antunes Davidᵃ, Leticia de Oliveiraᵃ, Mirtes Garcia Pereiraᵃ

Affiliation:

ᵃ Fluminense Federal University, Centro de Ciências Médicas, Departamento de Fisiologia.

Rua Hernani Mello 101, Dept Fisiologa, Laboratório de Neurofisiologia do Comportamento (LABNEC), Instituto Biomédico,

São Domingos, Niterói, RJ, Brazil.

CEP:24210130

ᵇ Federal University of Rio de Janeiro, Psychiatry Institute of the Federal University of Rio de Janeiro (UFRJ/IPUB), Laboratório Integrado de Pesquisas sobre o Estresse (LINPES).

Avenida Venceslau Brás, 71 – Campus da Praia Vermelha, Botafogo, Rio de Janeiro, RJ, Brazil. CEP:22290-140

***Supplemental Material***

**Table S1**

*Mean CTQ total scores and subscales scores (original sample)*

|  | Total sample (n=443) | Female (n=350) | Male (n=93) |
| --- | --- | --- | --- |
|  |  |  |  |
| Physical Abuse | 7.06 | 6.90 | 7.63 |
| Sexual Abuse | 6.19 | 6.29 | 5.83 |
| Emotional Abuse | 10.52 | 10.55 | 10.43 |
| Physical Neglect | 6.3 | 6.22 | 6.59 |
| Emotional Neglect | 9.45 | 9.26 | 10.14 |
| Total CTQ score | 39.52 | 39.23 | 40.62 |

In the following analysis, CTQ scores for each maltreatment were considered a continuous independent variable in the multivariate model for revictimization and PTSD symptom prediction.

**Table S2**

*The Impact of Childhood Maltreatment on Revictimization: Bivariate and Multivariate Negative Binomial Regression Model*

| Revictimization | Bivariate Model | | | Multivariate Model  (raw) | | | Multivariate Model  (adjusted for gender, age and socioeconomic status) | | |  |
| --- | --- | --- | --- | --- | --- | --- | --- | --- | --- | --- |
|  | *IRR* | 95% CI | *p-value* | *IRR* | 95% CI | *p-value* | *IRR* | 95% CI | *p-value* | |
| Physical Abuse | 1.0406 | [1.0214-1.0602] | 0.000 | 0.9975 | [0.9768-1.0186] | 0.816 | 0.9952 | [0.9747-1.0161] | 0.650 | |
| Sexual Abuse | 1.0387 | [1.0231-1.0546] | 0.000 | 1.0162 | [1.0007-1.0320] | 0.040 | 1.0114 | [0.9962-1.0269] | 0.142 | |
| Emotional Abuse | 1.0471 | [1.0357-1.0588] | 0.000 | 1.0428 | [1.0264-1.0594] | 0.000 | 1.0421 | [1.0260-1.0584] | 0.000 | |
| Physical Neglect | 1.0749 | [1.0480-1.1025] | 0.000 | 1.0335 | [1.0041-1.0638] | 0.025 | 1.0260 | [0.9970-1.0559] | 0.078 | |
| Emotional Neglect | 1.0355 | [1.0217-1.0496] | 0.000 | 0.9898 | [0.9716-1.0084] | 0.284 | 0.9913 | [0.9734-1.0096] | 0.353 | |

**Table S3**

*Predicting PTSD Symptoms: Bivariate and Multivariate Negative Binomial Regression for the Prediction of PTSD Severity*

| PTSD | Bivariate Model | | | | Multivariate Model | | Multivariate Model (adjusted) | | |  |
| --- | --- | --- | --- | --- | --- | --- | --- | --- | --- | --- |
|  | *IRR* | 95% CI | *p-value* | *IRR* | | 95% CI | *p-value* | *IRR* | 95% CI | *p-value* |
| Physical Abuse | 1.0705 | [1.0225-1.1208] | 0.004 | 0.9827 | | [0.9340-1.0340] | 0.502 | 0.9812 | [0.9324-1.0324] | 0.446 |
| Sexual Abuse | 1.0739 | [1.0347-1.1146] | 0.000 | 1.0406 | | [1.0021-1.0806] | 0.038 | 1.0388 | [1.0001-1.0789] | 0.049 |
| Emotional Abuse | 1.0890 | [1.0630-1.1156] | 0.000 | 1.0872 | | [1.0522-1.1233] | 0.000 | 1.0849 | [1.0498-1.1212] | 0.000 |
| Physical Neglect | 1.1086 | [1.0541-1.1659] | 0.000 | 1.0229 | | [0.9641-1.0853] | 0.452 | 1.0190 | [0.9611-1.0804] | 0.528 |
| Emotional Neglect | 1.0628 | [1.0337-1.0928] | 0.000 | 0.9841 | | [0.9490-1.0206] | 0.391 | 0.9866 | [0.9511-1.0235] | 0.474 |
